# Molecular alterations and potential actionable mutations in peritoneal mesothelioma: a scoping review of high-throughput sequencing studies

**DOI:** 10.1016/j.esmoop.2023.101600

**Published:** 2023-07-13

**Authors:** M.V. Dietz, J.P. van Kooten, M.S. Paats, J.G.V.J. Aerts, C. Verhoef, E.V.E. Madsen, H.J. Dubbink, J.H. von der Thüsen

**Affiliations:** 1 Departments of Surgical Oncology; 2 Pulmonary Medicine, Erasmus MC Cancer Institute, Rotterdam; 3 Department of Pathology, Erasmus MC, Rotterdam, The Netherlands

**Keywords:** molecular alterations, germline mutations, peritoneal mesothelioma, targeted therapies

## Abstract

**Background:**

Peritoneal mesothelioma (PeM) is a rare malignancy with a poor prognosis. Currently there is a lack of effective systemic therapies. Due to the rarity of PeM, it is challenging to study new treatment options. Off-label use of targeted drugs could be an effective approach. This scoping review aims to explore the genomic landscape of PeM to identify potential therapeutic targets.

**Materials and methods:**

A systematic literature search of Embase, Medline, Web of Science, the Cochrane Library, and Google Scholar was carried out up to 1 November 2022. Studies that reported on molecular alterations in PeM detected by high-throughput sequencing techniques were included. Genes that were altered in ≥1% of PeMs were selected for the identification of potential targeted therapies.

**Results:**

Thirteen articles were included, comprising 824 PeM patients. In total, 142 genes were altered in ≥1% of patients, of which 7 genes were altered in ≥10%. *BAP1* was the most commonly altered gene (50%). Other commonly altered genes were *NF2* (25%), *CDKN2A* (23%), *CDKN2B* (17%), *PBRM1* (15%), *TP53* (14%), and *SETD2* (13%). In total, 17% of PeM patients were carriers of a germline mutation, mainly in *BAP1* (7%).

**Conclusions:**

This scoping review provides an overview of the mutational landscape of PeM. Germline mutations might be a larger contributor to the incidence of PeM than previously thought. Currently available targeted therapy options are limited, but several targeted agents [such as poly (ADP-ribose) polymerase (PARP), enhancer of zeste homolog 2 (EZH2), and cyclin-dependent kinase 4/6 (CDK4/6) inhibitors] were identified that might provide new targeted therapy options in the future.

## Highlights

• This scoping review provides an overview of the mutational landscape of PeM.
• Germline mutations were present in 17% of patients and might be a larger contributor to PeM than previously thought.
• Based on the molecular signature, currently available targeted therapy options are limited.
• Several targeted agents such as PARP, EZH2, and CDK4/6 inhibitors might provide new targeted therapy options in the future.

## Introduction

Peritoneal mesothelioma (PeM) is a rare and aggressive malignancy. The prognosis of patients with PeM is very poor due to its non-specific clinical presentation, aggressive nature, and limited treatment options.^1^ Cytoreductive surgery combined with hyperthermic intraperitoneal chemotherapy could potentially cure a selected group of patients.^2^^,^^3^ About one-third of patients are eligible to undergo this extensive treatment and the recurrence rate is high.^1^ Currently available systemic therapies have limited efficacy, in the first-line as well as in the second-line or adjuvant setting.^4^, ^5^, ^6^, ^7^ Hence, there is a pressing need for new treatment options.

As PeM is a rare malignancy, it is challenging and extremely time-consuming to study these new treatment options, and to gather randomized evidence for treatment effectiveness. An effective approach could therefore be the off-label use of readily available targeted drugs. Currently, several trials are investigating such an approach, for example, the Dutch Drug Rediscovery Protocol (DRUP) trial.^8^ In this trial, patients with (solid) malignancies are treated with approved targeted agents using a personalized strategy by molecular profiling. A tailored approach is only feasible, however, if the tumor harbors actionable mutations to begin with.

Several studies reported on the mutational landscape of pleural mesothelioma (PM), the more common variant of mesothelioma, but studies on genetic alterations in PeM are scarce.^9^^,^^10^ Due to the rarity of PeM, most currently available therapies are extrapolated from PM. However, as these malignancies harbor important differences, such as sex distribution, age of onset, and asbestos exposure, it is likely that these diseases also present distinct molecular features.^10^, ^11^, ^12^ This scoping review aims to explore the genomic signature of PeM and its potential therapeutic targets.

## Methods

### Selection of literature

This scoping review was carried out (where possible) according to the ‘Preferred Reporting Items for Systematic Reviews and Meta-analysis Extension for Scoping Reviews’ (PRISMA-ScR) statement.^13^ A systematic search for available literature was carried out in the following databases: Embase (i.e. PubMed), Medline, Web of Science Core Collection, Cochrane Central Register of Controlled Trials, and Google Scholar (100-top ranked). The full search term per database is provided in Supplementary Table S1, available at https://doi.org/10.1016/j.esmoop.2023.101600. Databases were searched for articles published between the date of initiation and 1 October 2022.

For every record, title and abstract were screened by two independent reviewers (JPvK and MVD). Studies that reported on molecular alterations (i.e. gene mutations, gene fusions, and gene copy number variants) in mesothelioma, detected by high-throughput sequencing techniques, were selected for full text review. Animal studies, studies with cell lines, case reports, conference abstracts, papers without an available full (English) text, and studies that only included pleural or pericardial mesothelioma were excluded. Studies that only used RNA sequencing, comparative genomic hybridization, or targeted DNA sequencing of one specific gene were also excluded. In case of disagreement over studies to be included in this report, the study was discussed with a third reviewer (JHvdT).

### Data extraction and quality assessment

Due to a wide variety in methods used by different groups, meta-analyses were not considered feasible. The risk of bias was not assessed, due to the descriptive nature of the included reports. Data regarding the following patient characteristics were extracted from the included studies: sex, histology, tumor mutational burden (TMB), and gene alterations. Somatic as well as germline mutations were included. If data were not reported in the original article, it was reported as unknown. The included studies used various sequencing methods and different gene panels. The proportion of altered genes was based on the total number of patients included in articles that specifically tested for a particular gene. Only likely pathogenic genetic alterations were included, i.e. single nucleotide variants in oncogenes or tumor suppressor genes (TSGs), amplifications of oncogenes, oncogenic gene fusions, and complete loss of TSGs. Single copy number variations were not included. Genes were reported if they were altered in ≥1% of all patients and were investigated in at least 10% of the PeM cases. In addition, an overview of gene alterations (i.e. all types of alterations) present in ≥10% of PeMs that were investigated by whole exome (WES) or genome sequencing (WGS) was provided.

### Identification of targeted therapies

Genes that were altered in at least 1% of the patients were selected for identification of potential currently available targeted therapies. The selection of these therapies was based on the currently approved targeted therapies for solid malignancies by the European Medicines Agency (EMA) and targeted therapies that are available via the DRUP trial (NCT02925234).^14^^,^^15^ To gain insight into possible future perspectives, genes that were altered in ≥10% of the PeM samples were selected. Clinical trials investigating potential targeted therapies for these altered genes were identified with mycancergenome.org. Trials were selected in case they specifically included patients with solid tumors and alterations in one of the genes. Vaccine trials were excluded. Additional clinical trials specifically investigating targeted therapies in patients with PM were identified using ClinicalTrials.gov. A search was done for ‘Malignant Mesothelioma’, with the filter ‘Interventional studies’.

## Results

**Figure 1:**
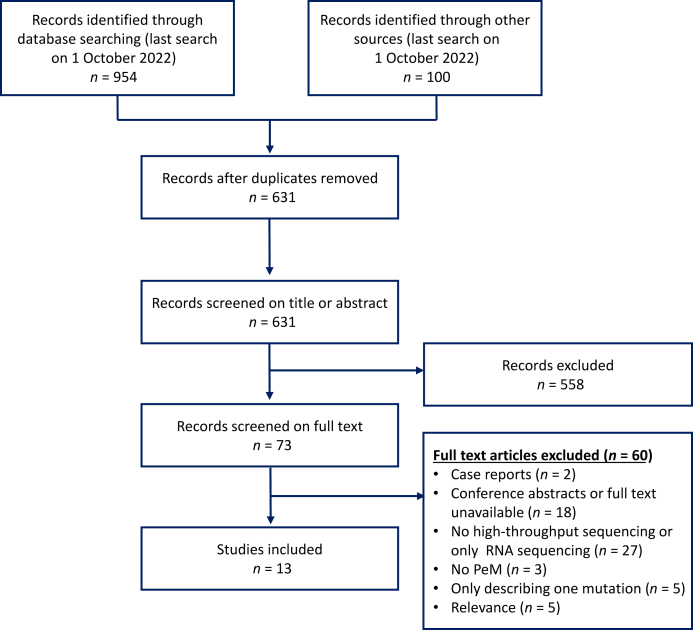
**Preferred Reporting Items for Systematic Reviews and Meta-analysis (PRISMA) flow diagram.** PeM, peritoneal mesothelioma.

Our search retrieved 631 records, of which 558 were excluded based on title/abstract screening (Figure 1). Full text screening was carried out for 73 records. A total of 13 articles were selected based on the inclusion and exclusion criteria (Table 1). Sequencing techniques that were used comprised (targeted-) next generation sequencing (NGS), WES, and WGS. Six out of 13 studies also analyzed blood, saliva, or normal tissue samples to identify germline mutations. The 13 included articles comprised 824 patients (Supplementary Table S2, available at https://doi.org/10.1016/j.esmoop.2023.101600). Data regarding gender were available for 746 patients, of which 347 (47%) were male. For 268 patients the histology type was reported, which was epithelioid in 233 (87%) of the cases.

**Table 1:** Overview of the included articles

	Reference	Year	*n*	Inclusion criteria	Sequencing method	Gene panel	Type of samples	Origin of tumor material
1	Sheffield et al.^16^	2015	2	NS	WGS	NA	FFPE tumor, blood	Diagnostic biopsy (*n* = 2), resection (*n* = 1)
2	Alakus et al.^17^	2015	7	Epithelioid PeM patients undergoing CRS	WES	NA	FFPE tumor, blood	Resection
3	Kato et al.^18^	2016	11	NS	NGS	Foundation Medicine	FFPE tumor	Unknown
4	Ugurluer et al.^19^	2016	4	NS	NGS	Foundation Medicine	FFPE tumor	Unknown
5	Joseph et al.^20^	2017	13	PeM limited to abdomen/pelvis	NGS	UCSF500 Cancer	FFPE tumor, FFPE normal tissue	Unknown
6	Panou et al.^21^	2018	10^a^ 17	Unrelated mesothelioma patients with germline mutations NS	NGS NGS	Targeted gene panel UCM-OncoPlus20,Foundation Medicine	FFPE tumor, blood, saliva FFPE tumor, blood, saliva	Unknown Unknown
7	Kim et al.^22^	2018	4	PeM patients treated with first-line palliative chemotherapy	NGS	OncoPanel version 2	FFPE tumor	Unknown
8	Shrestha et al.^23^	2019	18	Treatment-naïve PeM patients undergoing CRS	WES	NA	FFPE tumor, FFPE normal tissue or blood	Resection
9	Hung et al.^24^	2020	26	NS	NGS	Targeted gene panel	FFPE tumor	Resection (*n* = 21) or excisional biopsy (*n* = 5)
10	Taghizadeh et al.^25^	2020	3	Metastasized PeM refractory to standard treatment	NGS	Ion AmpliSeq Cancer Hotspot Panel v3	FFPE tumor	Unknown
11	Offin et al.^26^	2022	50	NS	NGS	MSK-IMPACT platform	Tumor, blood	Unknown
12	Dagogo-Jack et al.^12^	2022	314	Patients diagnosed with PeM	NGS	Foundation Medicine	FFPE tumor	Unknown
13	Hiltbrunner et al.^27^	2022	355	NS	NGS	Foundation Medicine	FFPE tumor	Unknown

CRS, cytoreductive surgery; FFPE, formalin-fixed paraffin-embedded; MSK-IMPACT, Memorial Sloan Kettering Cancer Center-IMPACT; NA, not applicable; NGS, next generation sequencing; NS, not specified; PeM, peritoneal mesothelioma; WES, whole exome sequencing; WGS, whole genome sequencing.

a Germline mutations.

### Gene alterations

**Figure 2:**
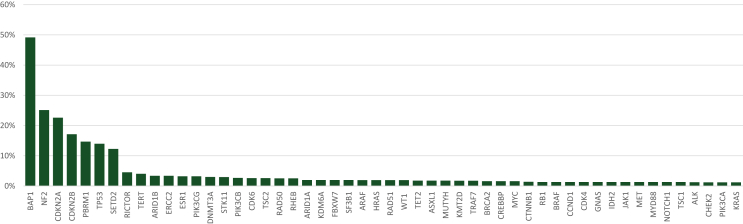
Gene alterations present in ≥1% of the peritoneal mesothelioma (PeM) patients.

A total of 52 genes (tested in at least 10% of the patients) harbored alterations in ≥1% of the patients (Figure 2). Of these, the most commonly altered genes were *BAP1* (49%), *NF2* (25%), *CDKN2A* (23%), *CDKN2B* (17%), *PBRM1* (15%), *TP53* (14%), and *SETD2* (12%). These gene alterations were not mutually exclusive. Simultaneous gene alterations were common in *CDKN2A* and *CDKN2B*, as well as in *BAP1*, *PBRM1*, and *SETD2*. WES or WGS was carried out in 27 patients. A total of 40 genes were mutated in ≥10% of these patients (i.e. ≥3 patients; Supplementary Figure S1, available at https://doi.org/10.1016/j.esmoop.2023.101600). A complete overview of all genes that were altered in ≥1% of the patients is provided in Supplementary Table S2, available at https://doi.org/10.1016/j.esmoop.2023.101600. Four studies reported on the TMB. Shrestha et al. only reported the highest (0.04 mutations/Mb) and the lowest TMB (0.001 mutations/Mb).^23^ Offin et al. and Dagogo-Jack et al. reported the median TMB for all patients with PeM, which was 1.8 mutations/Mb (range 0.0-14.9 mutations/Mb) and 1.25 mutations/Mb (interquartile range 0.00-1.25 mutations/Mb), respectively.^12^^,^^26^ Hiltbrunner et al. reported a high TMB (i.e. ≥10 mutations/Mb) in five patients (1.41%).^27^ Seven out of the 13 included articles also reported on PM. Table 2 provides an overview of the patient characteristics and the most common genomic alterations in PeM versus PM.

**Table 2:** Patient characteristics and genomic alterations in peritoneal versus pleural mesothelioma

	Peritoneal mesothelioma^a^	Pleural mesothelioma^b^
Median age range, years^c^	48-64	56-71
Male sex^d^	347 (47)	1490 (70)
Epithelioid histology^e^	233 (87)	2825 (71)
Germline mutations^f^	16 (17)	11 (7)
Altered genes		
*BAP1*	405 (49)	966 (44)
*NF2*	207 (25)	706 (32)
*CDKN2A*	186 (23)	1040 (48)^g^
*CDKN2B*	141 (17)	895 (42)^h^
*PBRM1*	120 (15)	145 (7)^i^
*TP53*	115 (14)	389 (18)
*SETD2*	101 (12)	217 (10)

PeM, peritoneal mesothelioma; PM, pleural mesothelioma.

a Total patients with PeM included, *n* = 824.

b Total patients with PM included, *n* = 2178.

c Reported for 420 patients with PeM, reported for 999 patients with PM.

d Reported for 739 patients with PeM, reported for 2118 patients with PM.

e Reported for 268 patients with PeM, reported for 395 patients with PM.

f Germline analysis carried out in 96 patients with PeM, carried out in 148 patients with PM.

g Reported for 1040 out of 2171 patients with PM.

h Reported for 895 out of 2134 patients with PM.

i Reported for 145 out of 2160 patients with PM.

### Germline mutations

Five out of 13 studies also reported on germline mutations specifically for PeM (Supplementary Table S2, available at https://doi.org/10.1016/j.esmoop.2023.101600). Sheffield et al. (*n* = 2) detected no germline mutations.^16^ Alakus et al. (*n* = 7) and Joseph et al. (*n* = 13) both identified one patient with a germline *BAP1* mutation.^17^^,^^20^ Offin et al. carried out germline testing for 30 out of 50 patients.^26^ Three patients harbored a germline mutation: *POT1* (*n* = 1), *MUTYH* (*n* = 1), and *BAP1* (*n* = 1). Panou et al. specifically screened unrelated mesothelioma patients for germline mutations.^21^ Out of 44 patients with PeM, 11 (25%) harbored a germline mutation. *BAP1* was the most frequently mutated gene in four patients (9%). Other mutated genes were *ATM*, *BRCA2*, *CDKN2A*, *CHEK2*, *MITF*, *SDHA*, and *WT1*, each reported in one patient. The patients with the *ATM* and *WT1* germline mutations also harbored a somatic *BAP1* mutation. One of the patients with a *BAP1* germline mutation also harbored a *MITF* germline variant of unknown significance (VUS) and a somatic *BAP1* (possibly second hit) and *CSF1R* mutation. Lastly, a germline *BAP1* VUS was detected in one patient. In total, 96 patients underwent germline testing, of whom 16 (17%) harbored a germline mutation, with *BAP1* germline mutations in 7 (7%) patients.

### Targeted therapies

Currently there are no targeted therapies available for patients with PeM and 1 of the 52 identified genes. For 12 out of these 52 genes (i.e. *ALK*, *BRCA2*, *CCND1*, *CDK4*, *CDK6*, *CDKN2A*, *CHEK2*, *GNAS*, *KRAS*, *MET*, *PIK3CA*, and *RAD50*), approved targeted therapies are available for other malignancies (Table 3). For *BRAF* mutations (i.e. V600E), there are also targeted agents available, but the gene alterations reported in the current review consisted of copy number gains for which these targeted agents are not indicated. Supplementary Table S3, available at https://doi.org/10.1016/j.esmoop.2023.101600, provides an overview of the targeted therapies and their approved indications or availability through the DRUP trial. Out of seven of the most commonly altered genes, for six genes, clinical trials were identified that investigate targeted agents for treatment of solid malignancies harboring alterations in these genes (Supplementary Table S4, available at https://doi.org/10.1016/j.esmoop.2023.101600).

**Table 3:** Genes with available targeted therapies for other malignancies

Gene	Alteration	Frequency in PeM	Targeted therapies Type	Targeted drugs Drug
*CDKN2A*	Loss/mutation	23%	CDK4/6 inhibitors	Palbociclib Ribociclib
*CDK6*	Amplification	3%	CDK4/6 inhibitors	Abemaciclib Palbociclib
*BRCA2*	Loss/mutation	2%	PARP inhibitors	Rucaparib
*CCND1*	Amplification	2%	CDK4/6 inhibitors	Abemaciclib Palbociclib
*ALK*	Fusion	1%	ALK inhibitors	Alectinib Crizotinib Lorlatinib
*CDK4*	Amplification	1%	CDK4/6 inhibitors	Abemaciclib Palbociclib Ribociclib
*CHEK2*	Mutation	1%	PARP inhibitors	Olaparib Talazoparib
*GNAS*	Mutation	1%	MEK inhibitors	Trametinib
*MET*	Amplification Fusion	1%	Multi-targeted receptor tyrosine kinase inhibitors	Crizotinib Cabozantinib
*PIK3CA*	Mutation	1%	PI3K inhibitors	Alpelisib
*KRAS*	G12C mutation	1%	KRAS inhibitors	Sotorasib

*ALK*, anaplastic lymphoma kinase; *CDK4/6*, cyclin-dependent kinases 4/6; *MEK*, mitogen-activated protein kinase kinase; *PARP*, poly (ADP-ribose) polymerase; PeM, peritoneal mesothelioma.

## Discussion

This scoping review aimed to provide an overview of the genomic landscape of PeM and its potential therapeutic targets, based on 13 studies comprising 824 patients with PeM. This review identified multiple gene alterations, present in various proportions of patients with PeM, reflecting a heterogeneous mutational landscape. *BAP1* was the most commonly mutated gene (49%). Other commonly affected genes were *NF2* (25%), *CDKN2A* (23%), *CDKN2B* (17%), *PBRM1* (15%), *TP53* (14%), and *SETD2* (13%). Interestingly, out of 96 patients who underwent germline testing, 16 (17%) were carriers of a germline mutation, mainly in *BAP1* (7%). Another significant proportion of cases might be caused by rarely occurring germline mutations in other genes. Germline mutations seem to be a larger contributor to the incidence of PeM than previously thought.

### Mutational landscape of PeM

The most common alterations in PeM were detected in TSGs. Inactivation of TSGs appears to play an important role in PeM development. Despite the heterogeneous mutational landscape of PeM, several pathways seem to be predominantly involved in PeM etiology.

#### DNA damage response

The DNA damage response (DDR) pathway is essential for genomic stability and defects in this pathway have been associated with the development of cancer. The current review shows that the DDR pathway also seems to be involved in PeM, which is in line with literature on mesothelioma.^28^ In almost half of the patients with PeM, *BAP1* was altered. *BAP1* is involved in multiple processes, including DDR, and acts as a TSG by binding to *BRCA1*, another well-known TSG.^29^ *BAP1* is located on chromosome 3p21, which is often lost in various malignancies.^30^ Other TSGs located on this locus are *SETD2* and *PBRM1*. Alterations of these genes were also frequently observed (12% and 15%, respectively) in the current review. Germline mutations in *BAP1* are known to cause a tumor predisposition syndrome, which is accompanied by the risk of early onset of several malignancies, such as (uveal) melanoma, renal cancer, and PeM.^31^ Other DDR-associated genes that were altered in PeM were *BRCA2*, *ERCC2*, and *RAD50/51*, all present in <5% of the samples.

#### Chromatin remodeling/DNA methylation

Chromatin remodeling and DNA methylation play an essential role in gene expression and alterations can contribute to the development of cancer. Epigenetic mechanisms also have an important function in the DDR, as reorganization of the chromatin structure is essential for DNA repair. The aforementioned *BAP1* gene performs its function in the DDR by binding to *BRCA1*, but also functions as a deubiquitinating enzyme, regulating chromatin remodeling. Another essential component of chromatin remodeling is the SWI/SNF complex.^32^ In the current review several SWI/SNF subunit genes were reported as altered in PeM, including *PBRM1* (15%), *ARID1B* (3%), and *ARID1A* (2%). Other genes involved in epigenetic gene regulation that were mutated in PeM are *DNMT3A*, *KDM6A*, *TET2*, *ASXL1*, *KMT2D*, and *IDH2*, all present in ≤3% of the PeM tumors.

#### Cell cycle regulation

Another pathway that seems to contribute to PeM development concerns cell cycle regulation. A key player in this pathway is *TP53*, a well-known TSG that encodes p53, and was mutated in 14% of the PeM samples. Other reported genes involved in cell cycle regulation are *CDKN2A/B*, *CDK4/6*, *CCND1*, *CHEK2*, and *Rb1*. *CDKN2A* was inactivated in 23% of PeMs and encodes for two tumor suppressor proteins, p16 and p14, which are both involved in the cell cycle regulation through inhibition of cyclin-dependent kinases 4/6 (*CDK4/6*) and stabilization of p53.^33^, ^34^, ^35^ Adjacent to *CDKN2A* lies *CDKN2B*, altered in 17% of cases, which encodes a cyclin-dependent kinase inhibitor (p15) that functions as a cell growth regulator that controls cell cycle G1 progression.^36^ Inactivation of *TP53* as well as *CDKN2A/B* is associated with a variety of malignancies.^33^^,^^37^

#### Kinase signaling pathways

Kinase signaling pathways are pivotal in cell growth and survival, and have been associated with the development of several malignancies.^38^^,^^39^ One of these pathways is the phosphoinositide 3-kinase/Protein kinase B/mammalian target of rapamycin (mTOR) signaling pathway. Genes that are involved in this pathway are *PIK3CA*, *PIK3CB*, *PIK3CG*, *RICTOR*, and *TSC1/2*, present in ≤5% of PeM cases. *NF2* is a TSG that encodes for the Merlin protein and is mainly involved in the Hippo pathway, but also impacts mTOR signaling.^40^ Alterations in *NF2* are known for causing the familial cancer predisposition syndrome neurofibromatosis type 2, but have also been associated with sporadic malignancies including mesothelioma, breast, colorectal, and renal cancers.^41^ *NF2* was altered in 25% of the PeMs, but no germline *NF2* mutations were observed. Lastly, the mitogen-activated protein kinase signaling pathway has been associated with a variety of tumors, but apparently does not play a major role in the development of PeMs as mutations in this pathway were less common (*HRAS* 2% and *KRAS* 1%).^42^

### PeM versus PM

Because PeM and PM are known to harbor differences in clinical characteristics, such as sex distribution, age of onset, and relation to asbestosis exposure, it was hypothesized that these differences might be reflected by the mutational landscape.^10^, ^11^, ^12^ Of the 13 included articles, 7 also reported on molecular alterations in PM (Table 2). Clinical characteristics between PeM and PM also seemed to differ in the studies that were included in the current review. Conforming to the large cohorts of Dagogo-Jack et al. and Hiltbrunner et al, the mutational landscapes of PM and PeM seem to be similar.^12^^,^^27^ However, lower prevalence of *CDKN2A/B* alterations was detected in PeM compared to PM, whereas *PBRM1* alterations were more common in PeM. The frequency of *BAP1* mutations in PM of 44% conforms to other studies reporting on the genetic landscape of PM and is similar to the 49% reported in patients with PeM.^9^^,^^12^^,^^43^^,^^44^ Other frequently altered genes in PeM such as *NF2* and *TP53* are also common in PM.^9^^,^^12^^,^^43^^,^^44^ Although rare, anaplastic lymphoma kinase (*ALK*) rearrangements were reported in 10 patients (1%) in the current review. This alteration seems to be more common in PeM, as very few cases of patients with PM with *ALK* rearrangements have been described.^12^^,^^27^^,^^45^, ^46^, ^47^

The current review showed that 17% of all patients with PeM who underwent germline testing harbored a germline mutation. Panou et al. was the only included study that also reported on germline mutations in PM (7%), but the proportion of germline mutations conforms to other studies reporting on mesothelioma in general, ranging from 0% to 8%.^21^^,^^48^, ^49^, ^50^, ^51^ This indicates that genetic predisposition plays a larger role in the development of PeM compared to PM. Subsequently, this might explain why the association between PeM and asbestosis exposure is less evident for PeM compared to the pleural variant, and hence contributes to the difference in sex distribution and age of onset. Several studies have highlighted these differences between PeM and PM, but the role of germline mutations in the etiology of PeM has been relatively underexposed.^52^^,^^53^ Further investigation should be done to unravel the role of germline mutations in PeM etiology.

### Targeted therapies

In the Netherlands, there are currently no approved targeted drugs for patients with PeM and one of the reported gene alterations. The loss and/or inactivation of TSGs appears to play an important role in PeM development. Targeting TSGs is known to be challenging and most of the currently available targeted drugs target oncogenes. In the past decades, the development of drugs targeting TSGs is increasing, resulting in potential new therapies for patients with PeM. The availability of these targeted therapies might be hampered by the rareness of PeM and its heterogeneous mutational landscape. Therefore, a ‘tailored approach’ with the off-label use of approved targeted drugs might be an effective strategy. This is not only relevant for PeM, but applies to other (rare) malignancies and provided the rationale for several multi-drug trials such as the Dutch DRUP trial (NCT02925234), the MATCH Screening Trial (NCT02465060) in the United States, the CAPTUR trial (NCT03297606) in Canada, and the ProTarget trial (NCT04341181) in Denmark.^54^

The Dutch DRUP trial consists of multiple arms, including one in which mesothelioma patients with *CDKN2A* loss or mutation (present in 23% of patients with PeM) were treated with ribociclib, a CDK4/6 inhibitor. Another arm included mesothelioma patients with a *PDGFRA* mutation, which, according to our data, has not been observed in PeM. Four of the trial arms included patients independent of tumor type and one of the reported alterations in PeM: olaparib [poly (ADP-ribose) polymerase (PARP) inhibitor] for alterations in DDR-related genes (*BRCA2*, *CHEK2*, and *RAD50*), trametinib (BRAF inhibitor) for *GNAS* mutations, and alectinib (ALK inhibitor) for *ALK* fusions. These alterations were rare in this review (present in ≤3%). A British trial with a similar approach specific for mesothelioma patients is the MiST trial (NCT03654833). This trial includes five treatment arms, including rucaparib (a PARP inhibitor) for patients with BRCA1/BAP1-deficient tumors and abemaciclib (a CDK4/6 inhibitor) in patients with p16ink4A-negative, CDKN2A-mutated tumors, which are more common in PeM. The first results of this trial showed that rucaparib and abemaciclib were both well tolerated and showed promising activity.^55^^,^^56^

Hopefully the outcomes of these multi-drug trials will support the rationale for a tailored approach resulting in more treatment options for patients with PeM. In addition to these multi-drug trials, several agents are available or under investigation for the treatment of other solid malignancies that target common genetic alterations in PeM (i.e. present in ≥10%). Below, a brief overview of targeted therapies that might be beneficial for PeM based on its molecular signature is provided (an overview of clinical trials is provided in Supplementary Table S4, available at https://doi.org/10.1016/j.esmoop.2023.101600).

#### PARP inhibitors

One of the targeted therapies that have been introduced in the past decade is PARP inhibitors, of which olaparib was the first approved inhibitor.^57^ *PARP* is involved in the DDR and inhibition of PARP results in the inability to correct DNA single strand breaks, leading to cell death in DDR-deficient cells. Assuming that DDR deficiencies are an important contributor to PeM development, PARP inhibitors might be a promising therapy for patients with PeM. PARP inhibitors are currently approved by the EMA for the treatment of several solid malignancies, including breast and ovarian cancer, and mutations in DDR-related genes such as *BRCA1/2*, *ATM*, and *CHEK2*. Due to the role of *BAP1* in DDR, it has been hypothesized that PARP inhibition might also be effective in the treatment of BAP1-altered tumors.^29^^,^^58^ In a recently published trial, 23 patients with mesothelioma (i.e. 16 with PM and 7 with PeM) were treated with olaparib, independent of mutational status.^59^ Unfortunately, olaparib had limited activity in patients with mesothelioma, including in patients with *BAP1* mutations. The MiST trial on the other hand showed that rucaparib showed promising activity in patients with BAP1-deficient mesothelioma.^55^ Currently, a phase II trial is investigating the effect of olaparib in patients with mesothelioma and a *BAP1* mutation (NCT04515836). Several other trials are currently investigating the efficacy of PARP inhibitors (i.e. niraparib, olaparib, talazoparib, and veliparib) in other solid tumors with *BAP1* mutations.

#### EZH2 inhibitors

Another targeted therapy of interest is enhancer of zeste homolog 2 (EZH2) inhibition due to its function in transcriptional activation and suppression of important TSGs. In mesothelioma, high expression of EZH2 has been reported, especially in patients with loss of *BAP1*.^60^ Tazemetostat is the first EZH2 inhibitor that received an orphan designation by the EMA. In a recently finished multicenter phase II trial, mesothelioma patients with loss of *BAP1* were treated with tazemetostat.^61^ This trial showed that this therapy was safe and antitumor activity was observed in more than half of the patients. Currently, a phase I/II trial is investigating another EZH2 inhibitor, CPI-0209, in patients with various malignancies with the loss of *BAP1*, including mesothelioma (NCT04104776).

#### CDK4/6 inhibitors

Due to the involvement of the cell cycle regulation pathway in PeM, another promising targeted therapy is CDK4/6 inhibition. Currently, CDK4/6 inhibitors (i.e. abemaciclib, palbociclib, and ribociclib) are approved by the EMA for the treatment of hormone receptor (HR)-positive and human epidermal growth factor receptor 2 (*HER2*)-negative breast cancer, independent of mutational profile. In the DRUP trial, patients with CDK4/6-amplified tumors are treated with CDK4/6 inhibitors.^14^ Although *CDK4/6* amplifications were rare in PeM (1% and 3%, respectively), these inhibitors might also be beneficial for the treatment of tumors with loss of *CDKN2A/B* (23% and 17% of PeM, respectively), as these encode for proteins that inhibit CDK4/6. Currently, many trials are investigating the efficacy of CDK4/6 inhibitors in patients with CDKN2A/B-altered tumors, either as monotherapy or in combination with other targeted therapies. Lastly, in the DRUP trial, treatment with CDK4/6 inhibitors is also provided to patients with *CCND1* amplifications, due to the interplay of *CCND1* with *CDK4*.

#### Other targeted therapies

Another popular target gene is *ALK*, as it is the driver oncogene in ∼5% of patients with non-small-cell lung cancer (NSCLC).^62^ Several ALK inhibitors have been approved for treatment of patients with NSCLC and *ALK* alterations, and there is evidence that these agents are beneficial for other ALK-rearranged malignancies.^63^, ^64^, ^65^ For patients with mutations in *PIK3CA* and HR-positive and HER2-negative breast cancer, alpelisib (a phosphoinositide 3-kinase inhibitor) is approved by the EMA and the DRUP trial provides this treatment for several other PIK3CA-mutated tumors (not for PeM).^14^ For patients with *NF2*, *SETD2*, or *TP53* alterations (all present in ≥10% of PeM cases), there are currently no approved targeted therapies, independent of type of tumor. However, several clinical trials are investigating various drugs targeting these genes, which might result in new treatment options in the future.

### Current clinical implications

The heterogeneous mutational landscape of PeM together with the limited treatment options provide a rationale for mutational analysis. Although there are currently no approved targeted therapies for patients with PeM, several therapies are available in a clinical trial setting and might become available for patients with PeM in the future. Comprehensive screening for genetic alterations might be considered to simultaneously test for high- as well as low-frequency altered genes, with limited additional costs. Although most of the currently approved drugs target genes which are rarely altered in PeM (e.g. *PIK3CA* and *ALK*, altered in 1%), these patients could gain substantial benefit from these therapies. As the availability of targeted agents changes over time, the indication of mutational analysis (i.e. broad spectrum or selective mutational analysis) should be regularly reconsidered.

Another approach could be to identify predictive factors for specific mutations to select patients with PeM who are most likely to harbor these alterations. For example, Hiltbrunner et al. identified subgroups of patients with mesothelioma according to gene alterations as some mutations do not appear to be mutually exclusive and often occur simultaneously.^27^ This subgroup identification might not only be relevant for treatment purposes, but might also have prognostic value. Hiltbrunner et al. suggested that patients with *CDKN2A* alteration only or patients with simultaneous *CDKN2A* and *BAP1* alterations had poor survival outcomes. Lastly, mutational analysis can not only be used for selection of targeted therapies, but can also be used for prediction of sensitivity to other therapies.^66^, ^67^, ^68^ Several genetic alterations have been associated with sensitivity to specific chemotherapeutic drugs or immunotherapy. For example, due to its role in DDR, loss of *BAP1* might enhance response to platinum and pemetrexed chemotherapy.^51^^,^^69^ In addition, TMB has been shown to be a predictive biomarker for the response to immunotherapy.^70^ TMB was low in most PeM tumors, which may indicate limited benefit of immunotherapy.^12^^,^^23^, ^26^, ^27^ However, recent studies using different techniques for TMB assessment unraveled higher rates of genomic alterations in mesothelioma.^71^^,^^72^ The value of both mutational analysis and TMB assessment as predictive biomarkers for chemo- and immunotherapy needs to be further investigated before they can be implemented in daily practice.

Lastly, these data provide a rationale for referral of patients with PeM to a clinical geneticist for germline testing, as germline mutations were present in a large proportion of patients (17%). Panou et al. reported that patients with mesothelioma and germline mutations were younger at the onset of disease, more often had a second cancer diagnosis, and had minimal known asbestos exposure.^21^ This conforms to other studies reporting on germline mutations in patients with mesothelioma in general and resulted in a recent addition of advice on germline testing in the Dutch mesothelioma guidelines.^73^^,^^74^ However, further research should be done to assess the involvement of germline mutations in the time of onset of PeM specifically.

### Limitations

This scoping review has some limitations, mainly due to the heterogeneity of the included studies and lack of relevant data. It is important to take into account that the included studies comprised various populations of patients with PeM (i.e. treatment-naive patients versus patients treated with palliative chemotherapy or surgery). Another contributing factor to the heterogeneity is the difference in DNA sequencing methods. Targeted NGS studies, exploring a specific set of genes based on recurrently altered genes, cannot be directly compared to WGS studies covering the whole genome. To process data from high-throughput sequencing analyses, a set of bioinformatics algorithms, referred to as a bioinformatics pipeline framework, is needed. These bioinformatics pipeline frameworks are needed to process and analyze sequencing data to identify clinically relevant genetic alterations and often vary between studies, resulting in varying sensitivity to detect genomic alterations. The same applies to the measurement of TMB, for which bioinformatics algorithms are also known to strongly influence the results.^70^

Not all of the included studies provided full mutational data, hampering good interpretation. Some studies did not report on the clinical significance of the detected alterations and some studies were very limited in clinical data. The current review only included likely pathogenic gene alterations (including homozygous losses and amplifications of oncogenes), therefore single copy number variants were excluded. However, some studies only reported on whether a copy number variation concerned a loss or a gain, but did not report any details on the depth of losses (homozygous versus allelic loss) or number of extra copies.

## Conclusions

This scoping review provides an overview of the genetic landscape of PeM and aimed to identify targeted therapies. Alterations in *BAP1* were most common and present in almost half of the patients. Up to 17% of patients were carriers of a germline mutation, most frequently in *BAP1*, which adds to the understanding of PeM etiology and provides a rationale for further research. Based on the molecular signature of PeM, currently available targeted therapy options are very limited. However, clinical trials as well as currently available targeted therapies for other malignancies were identified that might provide benefit to patients with PeM, supporting the rationale for molecular diagnostics.
