# Supplementary figures and images for: Molecular alterations and potential actionable mutations in peritoneal mesothelioma: a scoping review of high-throughput sequencing studies
Source: ESMO Open. 2023 Jul 13;8(4):101600. doi: 10.1016/j.esmoop.2023.101600 (PMC10368826; doi:10.1016/j.esmoop.2023.101600)

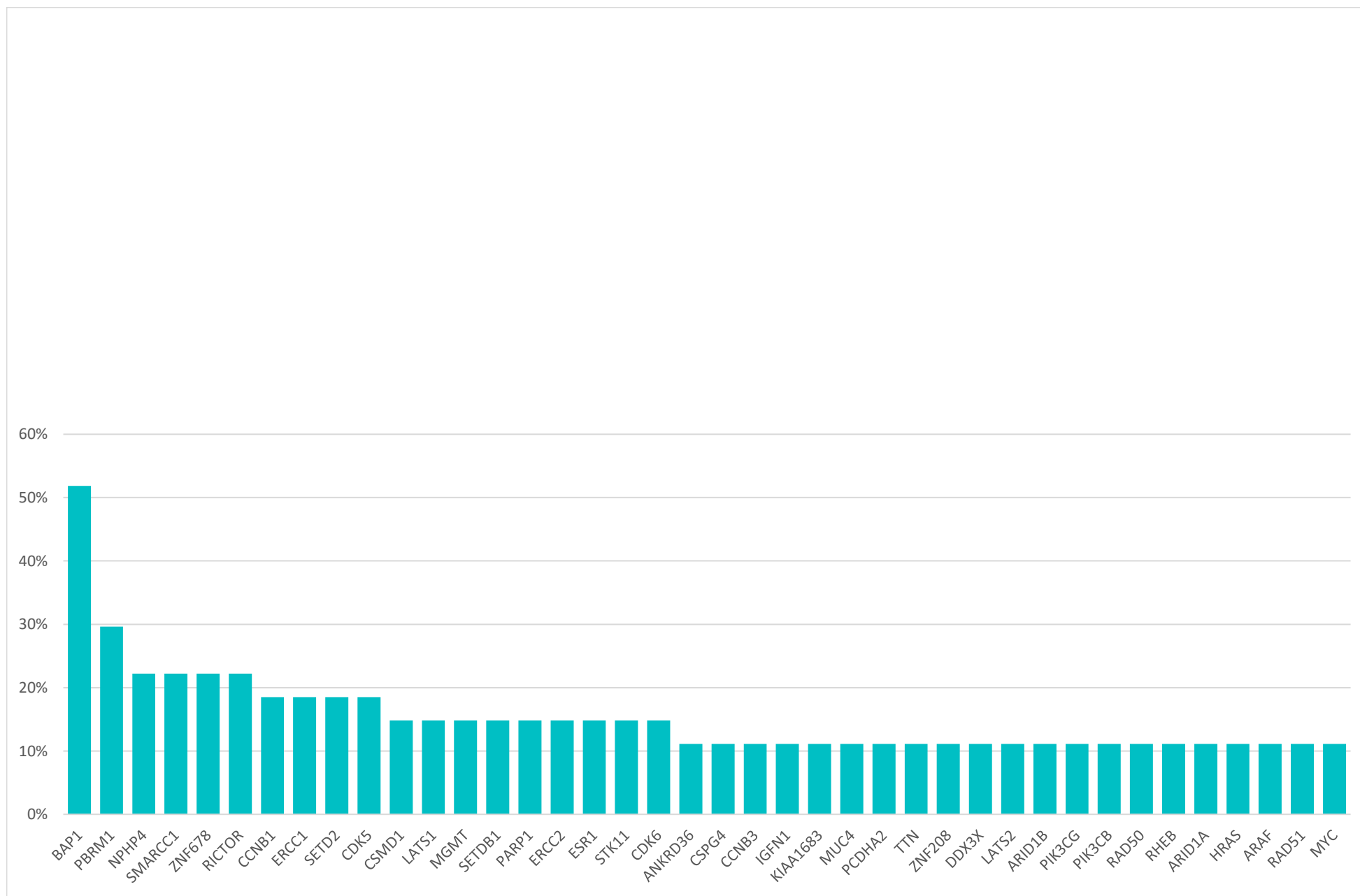

Supplement: Supplementary Figure 1 [file mmc1.pdf]
