# Supplementary material for: Molecular alterations and potential actionable mutations in peritoneal mesothelioma: a scoping review of high-throughput sequencing studies
Source: ESMO Open. 2023 Jul 13;8(4):101600. doi: 10.1016/j.esmoop.2023.101600 (PMC10368826; doi:10.1016/j.esmoop.2023.101600)
Supplement: Supplementary data [file mmc2.docx]

**Supplementary table 1.** Full search term per database

| **Database** | **Search term** |
| --- | --- |
| **Embase** | ('peritoneum mesothelioma'/de OR 'malignant peritoneal mesothelioma'/de OR ('mesothelioma'/de AND ('peritoneum tumor'/de OR 'peritoneum cancer'/de OR 'peritoneum'/de)) OR (((peritoneum* OR peritoneal*) NEAR/6 (mesotheliom*)) OR MPEM):ab,ti,kw) AND ('mutation'/exp OR 'copy number variation'/de OR 'comparative genomic hybridization'/de OR 'DNA polymorphism'/exp OR 'chromosome aberration'/exp OR 'gene rearrangement'/de OR 'genetic analysis'/exp OR 'protein expression'/de OR 'epidermal growth factor receptor'/de OR 'tumor mutational burden'/de OR 'sequence analysis'/exp OR 'genetic marker'/de OR 'gene translocation'/de OR 'genetic screening'/exp OR (mutation* OR mutant* OR ((gene OR genetic OR genes) NEAR/3 (alter* OR rearrang* OR re-arrang*)) OR mutagen* OR deletion* OR ((copy) NEAR/3 (number*) NEAR/3 (variat*)) OR ((compar*) NEAR/3 (genom*) NEAR/3 (hybrid*)) OR ((DNA OR gene* OR single-nucleotid*) NEAR/3 (polymorphism*)) OR ((chromosom*) NEAR/3 (abberat* OR instabil* OR abnormal* OR anomal* OR error* OR defect*)) OR ((genetic OR gene OR genome* OR sequenc*) NEAR/3 (analys*)) OR ((protein*) NEAR/3 (expression*)) OR ((epidermal*) NEAR/3 (growth-factor*) NEAR/3 (recept*)) OR egf-receptor* OR ((gene OR genetic) NEAR/3 (marker* OR transloc* OR screening OR testing))):ab,ti,kw) NOT ([Conference Abstract]/lim AND [1800-2017]/py) |
| **Medline** | (((exp Neoplasms, Mesothelial OR Mesothelioma, Malignant.rs) AND (Peritoneum/ OR Peritoneal Neoplasms/)) OR (((peritoneum* OR peritoneal*) ADJ6 (mesotheliom*)) OR MPEM).ab,ti,kf.) AND (exp Mutation/ OR exp Polymorphism, Genetic/ OR Comparative Genomic Hybridization/ OR DNA polymorphism/ OR exp Chromosome Aberrations/ OR exp Gene Rearrangement/ OR Genetic Testing/ OR Gene Expression/ OR exp ErbB Receptors/ OR exp Sequence Analysis/ OR Genetic Markers/ OR Translocation, Genetic/ OR (mutation* OR mutant* OR ((gene OR genetic OR genes) ADJ3 (alter* OR rearrang* OR re-arrang*)) OR mutagen* OR deletion* OR ((copy) ADJ3 (number*) ADJ3 (variat*)) OR ((compar*) ADJ3 (genom*) ADJ3 (hybrid*)) OR ((DNA OR gene* OR single-nucleotid*) ADJ3 (polymorphism*)) OR ((chromosom*) ADJ3 (abberat* OR instabil* OR abnormal* OR anomal* OR error* OR defect*)) OR ((genetic OR gene OR genome* OR sequenc*) ADJ3 (analys*)) OR ((protein*) ADJ3 (expression*)) OR ((epidermal*) ADJ3 (growth-factor*) ADJ3 (recept*)) OR egf-receptor* OR ((gene OR genetic) ADJ3 (marker* OR transloc* OR screening OR testing))).ab,ti,kf.) NOT (news OR congres* OR abstract* OR book* OR chapter* OR dissertation abstract*.pt. AND 1800:2017.(sa_year).) |
| **Cochrane** | ((((peritoneum* OR peritoneal*) NEAR/6 (mesotheliom*)) OR MPEM):ab,ti,kw) AND ( (mutation* OR mutant* OR ((gene OR genetic OR genes) NEAR/3 (alter* OR rearrang* OR re NEXT arrang*)) OR mutagen* OR deletion* OR ((copy) NEAR/3 (number*) NEAR/3 (variat*)) OR ((compar*) NEAR/3 (genom*) NEAR/3 (hybrid*)) OR ((DNA OR gene* OR single NEXT nucleotid*) NEAR/3 (polymorphism*)) OR ((chromosom*) NEAR/3 (abberat* OR instabil* OR abnormal* OR anomal* OR error* OR defect*)) OR ((genetic OR gene OR genome* OR sequenc*) NEAR/3 (analys*)) OR ((protein*) NEAR/3 (expression*)) OR ((epidermal*) NEAR/3 (growth NEXT factor*) NEAR/3 (recept*)) OR egf NEXT receptor* OR ((gene OR genetic) NEAR/3 (marker* OR transloc* OR screening OR testing))):ab,ti,kw) |
| **Web of Science** | TS=(((((peritoneum* OR peritoneal*) NEAR/5 (mesotheliom*)) OR MPEM)) AND ( (mutation* OR mutant* OR ((gene OR genetic OR genes) NEAR/2 (alter* OR rearrang* OR re-arrang*)) OR mutagen* OR deletion* OR ((copy) NEAR/2 (number*) NEAR/2 (variat*)) OR ((compar*) NEAR/2 (genom*) NEAR/2 (hybrid*)) OR ((DNA OR gene* OR single-nucleotid*) NEAR/2 (polymorphism*)) OR ((chromosom*) NEAR/2 (abberat* OR instabil* OR abnormal* OR anomal* OR error* OR defect*)) OR ((genetic OR gene OR genome* OR sequenc*) NEAR/2 (analys*)) OR ((protein*) NEAR/2 (expression*)) OR ((epidermal*) NEAR/2 (growth-factor*) NEAR/2 (recept*)) OR egf-receptor* OR ((gene OR genetic) NEAR/2 (marker* OR transloc* OR screening OR testing)))) ) AND DT=(Article OR Review OR Letter OR Early Access) |
| **Google Scholar** | 'peritoneum\|peritoneal mesothelioma\|mesotheliomas'\|MPEM mutation\|mutant\|mutations\|'gene\|genetic\|genes\|DNA\|genome\|sequence alteration\|rearrangement\|deletion\|polymorphism\|abberation\|instability\|abnormality\|anomaly\| error\|defect\|marker\|translocation' |

**Supplementary table 2.** Patient-/tumor characteristics and gene aberrations per article

| Author | Sheffield | Alakus | Kato | Ugurluer | Joseph | Panou | Kim | Shreshta | Hung | Taghizadeh | Offin | Dagago | Hiltbrunner | Total n(%) |  |
| --- | --- | --- | --- | --- | --- | --- | --- | --- | --- | --- | --- | --- | --- | --- | --- |
| Patient/tumor characteristics | | | | | | | | | | | | | | | |
| Number of patients (n) | 2 | 7 | 11 | 4 | 13 | 17 | 4 | 18 | 26 | 3 | 50 | 314 | 355 | **824 (100)** |  |
| Epithelioid (n) | 1 | 7 | NR | 2 | 12 | 17 | NR | 18 | 23 | 2 | 45 | 106 ^a^ | NR | 233 (87) |  |
| Male (n) | 0 | NR | NR | 4 | 5 | NR | 2 | 7 | 10 | 1 | NR | 150 | 168 | 347 (47) |  |
| Age (years, median) | NR | NR | NR | 63 | 62 | NR | 58 | NR | 61 | 48 | 62 | 64 | NR | 58 – 63 ^b^ |  |
| Asbestosis exposure | 0 | NR | NR | NR | NR | NR | NR | 1 | 8 | NR | NR | NR | NR | 9 (23) |  |
| Tumor mutational burden (mutations/Mb) | NR | NR | NR | NR | NR | NR | NR | NR | NR | NR | 1.8 (range 0.0 -14.9) | 1.25 (IQR 0.0 – 2.50) | NR ^c^ | 1.25 – 1.8 ^b^ |  |
| Germline mutational analysis | | | | | | | | | | | | | | | |
| Germline analysis performed (n) | 2 | 7 | ND | ND | 13 | 44 | ND | ND | ND | ND | 30 | ND | ND | **96 (100)** |  |
| Germline aberration (n)  BAP1  POT1  MUTYH  ATM  BRCA2  CDKN2A  CHEK2  MITF  SDHA  WT1 |  | 1 |  |  | 1 | 4      1  1  1  1  1  1  1 |  |  |  |  | 1  1  1 |  |  | 7 (7)  1 (1)  1 (1)  1 (1)  1 (1)  1 (1)  1 (1)  1 (1)  1 (1)  1 (1) |  |
| Total |  | 1 |  |  | 1 | 11 |  |  |  |  | 3 |  |  | **16 (17)** |  |
| Gene aberrations in genes included in the gene panels of ≥ 10% of the patients | | | | | | |  | | | | | | | |  |
| BAP1 | 0 | 3 | 3 | 1 | 9 | 11 | 1 | 11 | 14 | 1 | 30 | 151 | 170 | 405 (49) |  |
| NF2 | 2 | 0 | 4 | 1 | 3 | 3 | 0 | 0 | 2 | 1 | 12 | 85 | 94 | 207 (25) |  |
| CDKN2A | 1 | 0 | 2 | 0 | 1 | 1 | 0 | 0 | 3 | 0 | 4 | 82 | 92 | 186 (23) |  |
| CDKN2B | 0 | 0 | 1 | 0 | 1 | 0 | 0 | 0 | 3 | 0 | 4 | 63 | 69 | 141 (17) |  |
| PBRM1 | 0 | 0 | 0 | 0 | 0 | 2 | 0 | 8 | 5 |  | 0 | 50 | 55 | 120 (15) |  |
| TP53 | 1 | 0 | 1 | 0 | 0 | 0 | 0 | 1 | 3 | 1 | 8 | 47 | 53 | 115 (14) |  |
| SETD2 | 0 | 0 | 0 | 2 | 2 | 3 | 0 | 5 | 5 | 1 | 11 | 35 | 37 | 101 (12) |  |
| RICTOR | 0 | 0 | 1 | 0 | 0 | 0 | 0 | 6 | 0 | 0 | 0 | # | # | 7 (5) |  |
| TERT | 0 | 0 | 0 | 0 | 1 | 1 | 0 | 0 | 0 | 0 | 2 | # | 15 | 19 (4) |  |
| ARID1B | 0 | 0 |  |  | 0 |  |  | 3 | 1 | 0 | 0 |  |  | 4 (3) |  |
| ERCC2 | 0 | 0 |  |  | 0 |  |  | 4 | 0 | 0 | 0 | # | # | 4 (3) |  |
| ESR1 | 0 | 0 | 0 | 1 | 0 | 0 | 0 | 4 | 0 | 0 | 0 | # | # | 5 (3) |  |
| PIK3CG | 0 | 0 |  |  | 0 |  | 0 | 3 |  |  | 0 |  |  | 3 (3) |  |
| DNMT3A | 0 | 0 | 1 | 0 | 0 | 0 | 0 | 0 | 0 |  | 0 | # | 14 | 15 (3) |  |
| STK11 | 0 | 0 | 0 | 0 | 0 | 0 | 0 | 4 | 0 | 0 | 2 | # | 9 | 15 (3) |  |
| PIK3CB | 0 | 0 | 0 | 0 |  | 0 |  | 3 |  | 0 | 0 | # | # | 3 (3) |  |
| CDK6 | 0 | 0 | 0 | 0 | 0 | 0 | 0 | 4 | 0 | 0 | 0 | # | # | 4 (3) |  |
| TSC2 | 0 | 0 | 1 | 0 | 0 | 0 | 0 | 0 | 3 | 0 | 0 | # | # | 4 (3) |  |
| RAD50 | 0 | 0 |  |  | 0 |  |  | 3 | 0 | 0 | 0 |  |  | 3 (3) |  |
| RHEB | 0 | 0 |  |  | 0 |  |  | 3 | 0 | 0 | 0 |  |  | 3 (3) |  |
| KDM6A | 0 | 0 | 0 | 1 | 0 | 1 | 0 | 1 | 0 |  | 0 | # | # | 3 (2) |  |
| ARID1A | 0 | 0 | 0 | 0 | 1 | 0 | 0 | 3 | 0 | 0 | 0 | # | 6 | 10 (2) |  |
| FBXW7 | 0 | 0 | 0 | 0 | 0 | 1 | 0 | 0 | 0 | 0 | 0 | # | 9 | 10 (2) |  |
| SF3B1 | 0 | 0 | 0 | 0 | 0 | 0 | 0 | 1 | 0 | 0 | 0 | # | 9 | 10 (2) |  |
| ARAF | 0 | 0 | 0 | 0 | 0 | 0 | 0 | 3 | 0 | 0 | 0 | # | # | 3 (2) |  |
| HRAS | 0 | 0 | 0 | 0 | 0 | 0 | 0 | 3 | 0 | 0 | 0 | # | # | 3 (2) |  |
| RAD51 | 0 | 1 | 0 | 0 | 0 | 0 | 0 | 2 | 0 | 0 | 0 | # | # | 3 (2) |  |
| WT1 | 0 | 0 | 0 | 0 | 1 | 1 | 0 | 0 | 1 | 0 | 0 | # | # | 3 (2) |  |
| TET2 | 0 | 0 | 0 | 0 | 0 | 0 | 0 | 0 | 0 |  | 0 | # | 9 | 9 (2) |  |
| ASXL1 | 0 | 0 | 0 | 1 | 0 | 0 | 0 | 0 | 0 | 0 | 0 | # | 8 | 9 (2) |  |
| MUTYH | 0 | 0 | 0 | 0 | 0 | 0 | 0 | 0 | 0 |  | 2 | # | 7 | 9 (2) |  |
| KMT2D | 0 | 0 | 0 | 0 | 0 | 0 | 0 | 1 | 0 |  |  | # | 7 | 8 (2) |  |
| TRAF7 | 0 | 0 |  |  | 0 |  |  | 1 | 1 |  | 0 |  |  | 2 (2) |  |
| BRCA2 | 0 | 0 | 1 | 0 | 0 | 0 | 0 | 1 | 0 | 0 | 0 | # | 6 | 8 (2) |  |
| CREBBP | 0 | 0 | 0 | 0 | 0 | 0 | 0 | 0 | 0 | 0 | 2 | # | 6 | 8 (2) |  |
| MYC | 0 | 1 | 0 | 0 | 0 | 0 | 0 | 2 | 0 | 0 | 0 | # | 5 | 8 (2) |  |
| CTNNB1 | 0 | 0 | 0 | 0 | 0 | 0 | 0 | 0 | 0 | 0 | 0 | # | 7 | 7 (1) |  |
| RB1 | 0 | 0 | 0 | 0 | 0 | 0 | 0 | 1 | 0 | 0 | 0 | # | 5 | 6 (1) |  |
| BRAF | 0 | 0 | 0 | 0 | 0 | 0 | 0 | 2 | 0 | 0 | 0 | # | # | 2 (1) |  |
| CCND1 | 0 | 0 | 0 | 0 | 0 | 0 | 0 | 2 | 0 | 0 | 0 | # | # | 2 (1) |  |
| CDK4 | 0 | 0 | 0 | 0 | 0 | 0 | 0 | 2 | 0 | 0 | 0 | # | # | 2 (1) |  |
| GNAS | 0 | 0 | 0 | 0 | 0 | 0 | 0 | 2 | 0 | 0 | 0 | # | # | 2 (1) |  |
| IDH2 | 0 | 0 | 0 | 0 | 0 | 0 | 0 | 2 | 0 | 0 | 0 | # | # | 2 (1) |  |
| JAK1 | 0 | 0 | 0 | 2 | 0 | 0 | 0 | 0 | 0 | 0 | 0 | # | # | 2 (1) |  |
| MET | 0 | 0 | 1 | 0 | 1 | 0 | 0 | 0 | 0 | 0 | 0 | # | # | 2 (1) |  |
| MYD88 | 0 | 0 | 0 | 0 | 0 | 0 | 0 | 0 | 0 | 0 | 2 | # | # | 2 (1) |  |
| NOTCH1 | 1 | 0 | 0 | 0 | 0 | 1 | 0 | 0 | 0 | 0 | 0 | # | # | 2 (1) |  |
| TSC1 | 0 | 0 | 0 | 1 | 0 | 0 | 0 | 0 | 1 | 0 | 0 | # | # | 2 (1) |  |
| ALK | 0 | 0 | 1 | 0 | 0 | 0 | 0 | 0 | 3 | 0 | 0 | 2 | 4 | 10 (1) |  |
| CHEK2 | 0 | 0 | 0 | 0 | 0 | 0 | 0 | 0 | 0 | 0 | 0 | # | 6 | 6 (1) |  |
| KRAS | 0 | 0 | 1 | 0 | 0 | 0 | 0 | 0 | 0 | 0 | 0 | # | 5 | 6 (1) |  |
| PIK3CA | 0 | 0 | 0 | 0 | 0 | 1 | 0 | 1 | 0 | 0 | 0 | # | 4 | 6 (1) |  |
| Other gene aberrations present in ≥ 1% of the patients | | | | | | | | | | | | | | | |
| NPHP4 | 0 | 0 |  |  |  |  |  | 6 |  |  |  |  |  | 6 (22) |  |
| SMARCC1 | 0 | 0 |  |  |  |  |  | 6 |  |  |  | # | # | 6 (22) |  |
| ZNF678 | 0 | 0 |  |  |  |  |  | 6 |  |  |  | # | # | 6 (22) |  |
| CCNB1 | 0 | 1 |  |  |  |  |  | 4 |  |  |  | # | # | 5 (19) |  |
| ERCC1 | 0 | 0 |  |  |  |  |  | 5 |  |  |  | # | # | 5 (19) |  |
| CSMD1 | 1 | 1 |  |  |  |  |  | 2 |  |  |  | # | # | 4 (15) |  |
| LATS1 | 0 | 0 |  |  |  |  |  | 4 |  |  |  | # | # | 4 (15) |  |
| MGMT | 0 | 0 |  |  |  |  |  | 4 |  |  |  | # | # | 4 (15) |  |
| SETDB1 | 1 | 0 |  |  |  |  |  | 3 |  |  |  | # | # | 4 (15) |  |
| ANKRD36 | 1 | 0 |  |  |  |  |  | 2 |  |  |  |  |  | 3 (11) |  |
| CCNB3 | 0 | 0 |  |  |  |  |  | 3 |  |  |  |  |  | 3 (11) |  |
| CSPG4 | 1 | 0 |  |  |  |  |  | 2 |  |  |  |  |  | 3 (11) |  |
| IGFN1 | 1 | 1 |  |  |  |  |  | 1 |  |  |  |  |  | 3 (11) |  |
| KIAA1683 | 1 | 1 |  |  |  |  |  | 1 |  |  |  |  |  | 3 (11) |  |
| MUC4 | 1 | 0 |  |  |  |  |  | 2 |  |  |  |  |  | 3 (11) |  |
| PCDHA2 | 0 | 1 |  |  |  |  |  | 2 |  |  |  |  |  | 3 (11) |  |
| TTN | 1 | 0 |  |  |  |  |  | 2 |  |  |  |  |  | 3 (11) |  |
| ZNF208 | 1 | 0 |  |  |  |  |  | 2 |  |  |  |  |  | 3 (11) |  |
| DDX3X | 0 | 1 |  |  | 2 | 1 |  | 2 |  | 0 |  |  |  | 6 (10) |  |
| CDK5 | 0 | 0 |  |  |  |  |  | 5 | 0 |  |  | # | # | 5 (9) |  |
| LATS2 | 1 | 0 |  |  |  |  |  | 2 |  |  | 4 |  |  | 7 (9) |  |
| ABCA7 | 0 | 1 |  |  |  |  |  | 1 |  |  |  |  |  | 2 (7) |  |
| BZW2 | 0 | 0 |  |  |  |  |  | 2 |  |  |  |  |  | 2 (7) |  |
| CD320 | 0 | 1 |  |  |  |  |  | 1 |  |  |  |  |  | 2 (7) |  |
| CLPX | 1 | 0 |  |  |  |  |  | 1 |  |  |  |  |  | 2 (7) |  |
| CNTNAP3B | 2 | 0 |  |  |  |  |  | 0 |  |  |  |  |  | 2 (7) |  |
| CYLC1 | 0 | 0 |  |  |  |  |  | 2 |  |  |  |  |  | 2 (7) |  |
| CYSLTR1 | 0 | 1 |  |  |  |  |  | 1 |  |  |  |  |  | 2 (7) |  |
| DMXL1 | 0 | 1 |  |  |  |  |  | 1 |  |  |  |  |  | 2 (7) |  |
| DYSF | 1 | 0 |  |  |  |  |  | 1 |  |  |  |  |  | 2 (7) |  |
| E2F1 | 0 | 0 |  |  |  |  |  | 2 |  |  |  |  |  | 2 (7) |  |
| E2F8 | 1 | 0 |  |  |  |  |  | 1 |  |  |  |  |  | 2 (7) |  |
| EIF4G2 | 0 | 0 |  |  |  |  |  | 2 |  |  |  |  |  | 2 (7) |  |
| EPHB2 | 0 | 1 |  |  |  |  |  | 1 |  |  |  |  |  | 2 (7) |  |
| EVI5L | 1 | 0 |  |  |  |  |  | 1 |  |  |  |  |  | 2 (7) |  |
| GPR179 | 1 | 0 |  |  |  |  |  | 1 |  |  |  |  |  | 2 (7) |  |
| GSE1 | 0 | 0 |  |  |  |  |  | 2 |  |  |  |  |  | 2 (7) |  |
| HDAC7 | 1 | 0 |  |  |  |  |  | 1 |  |  |  |  |  | 2 (7) |  |
| HSPG2 | 0 | 0 |  |  |  |  |  | 2 |  |  |  |  |  | 2 (7) |  |
| KCTD4 | 0 | 1 |  |  |  |  |  | 1 |  |  |  |  |  | 2 (7) |  |
| KRTAP1-1 | 0 | 0 |  |  |  |  |  | 2 |  |  |  |  |  | 2 (7) |  |
| MACF1 | 1 | 0 |  |  |  |  |  | 1 |  |  |  |  |  | 2 (7) |  |
| MOB1B | 0 | 0 |  |  |  |  |  | 2 |  |  |  |  |  | 2 (7) |  |
| MUC17 | 0 | 0 |  |  |  |  |  | 2 |  |  |  |  |  | 2 (7) |  |
| MUC5B | 1 | 0 |  |  |  |  |  | 1 |  |  |  |  |  | 2 (7) |  |
| MYBPC1 | 0 | 1 |  |  |  |  |  | 1 |  |  |  |  |  | 2 (7) |  |
| NEFH | 0 | 0 |  |  |  |  |  | 2 |  |  |  |  |  | 2 (7) |  |
| OBSCN | 1 | 0 |  |  |  |  |  | 1 |  |  |  |  |  | 2 (7) |  |
| PKHD1 | 1 | 1 |  |  |  |  |  | 0 |  |  |  |  |  | 2 (7) |  |
| PLXNB2 | 1 | 0 |  |  |  |  |  | 1 |  |  |  |  |  | 2 (7) |  |
| PPP1R13B | 0 | 0 |  |  |  |  |  | 2 |  |  |  |  |  | 2 (7) |  |
| PRG4 | 0 | 0 |  |  |  |  |  | 2 |  |  |  |  |  | 2 (7) |  |
| RAB12 | 0 | 1 |  |  |  |  |  | 1 |  |  |  |  |  | 2 (7) |  |
| RASA3 | 1 | 0 |  |  |  |  |  | 1 |  |  |  |  |  | 2 (7) |  |
| SALL3 | 0 | 0 |  |  |  |  |  | 2 |  |  |  |  |  | 2 (7) |  |
| SEZ6L2 | 0 | 1 |  |  |  |  |  | 1 |  |  |  |  |  | 2 (7) |  |
| STARD9 | 0 | 1 |  |  |  |  |  | 1 |  |  |  |  |  | 2 (7) |  |
| SYNE3 | 0 | 1 |  |  |  |  |  | 1 |  |  |  |  |  | 2 (7) |  |
| TENM3 | 0 | 0 |  |  |  |  |  | 2 |  |  |  |  |  | 2 (7) |  |
| TRPM3 | 1 | 0 |  |  |  |  |  | 1 |  |  |  |  |  | 2 (7) |  |
| USP9X | 0 | 0 |  |  |  |  |  | 2 |  |  |  |  |  | 2 (7) |  |
| WWC1 | 0 | 0 |  |  |  |  |  | 2 |  |  |  |  |  | 2 (7) |  |
| YWHAB | 0 | 0 |  |  |  |  |  | 2 |  |  |  |  |  | 2 (7) |  |
| ZHX3 | 0 | 1 |  |  |  |  |  | 1 |  |  |  |  |  | 2 (7) |  |
| ZNF318 | 0 | 1 |  |  |  |  |  | 1 |  |  |  |  |  | 2 (7) |  |
| ZNF626 | 1 | 0 |  |  |  |  |  | 1 |  |  |  |  |  | 2 (7) |  |
| ZNF814 | 0 | 0 |  |  |  |  |  | 2 |  |  |  |  |  | 2 (7) |  |
| PARP1 | 1 | 0 | 0 | 0 |  | 0 |  | 3 |  |  |  | # | # | 4 (7) |  |
| MUC2 | 1 | 1 |  |  |  |  | 0 | 0 |  |  |  |  |  | 2 (6) |  |
| PIK3R2 | 0 | 0 |  |  | 0 |  |  | 2 |  |  |  |  |  | 2 (5) |  |
| WWTR1 | 0 | 0 |  |  | 0 |  |  | 2 |  |  |  |  |  | 4 (5) |  |
| XRCC1 | 0 | 0 |  |  |  |  |  | 2 | 0 |  |  |  |  | 2 (4) |  |
| PRMT1 | 0 | 0 |  |  |  |  |  | 1 |  |  |  |  |  | 1 (4) |  |
| SETDB2 | 0 | 0 |  |  |  |  |  | 1 |  |  |  |  |  | 1 (4) |  |
| FANCD2 | 0 | 0 |  |  |  |  | 0 | 1 | 0 | 0 |  |  |  | 1 (2) |  |
| PIK3R3 | 0 | 0 |  |  |  |  |  | 1 |  |  | 0 |  |  | 1 (1) |  |

IQR= interquartile range, ND= not determined, NR= not reported or not specified, TMB= tumor mutational burden

^a^ Dagogo-Jack et al. reported on histologic typing in 128 patients

^b^ Range

^c^ Hiltbrunner et al. reported 5 patients (1.41%) with PeM with a high TMB (i.e. ≥10 mutations/Mb)

# Gene was included in the gene panel, but not reported as the frequency of alterations were ≤10% for Dagogo et al. and ≤2% for Hiltbrunner et al.

**Supplementary figure 1.** Gene aberrations present in ≥10% of the PeM patients that underwent WGS/WES
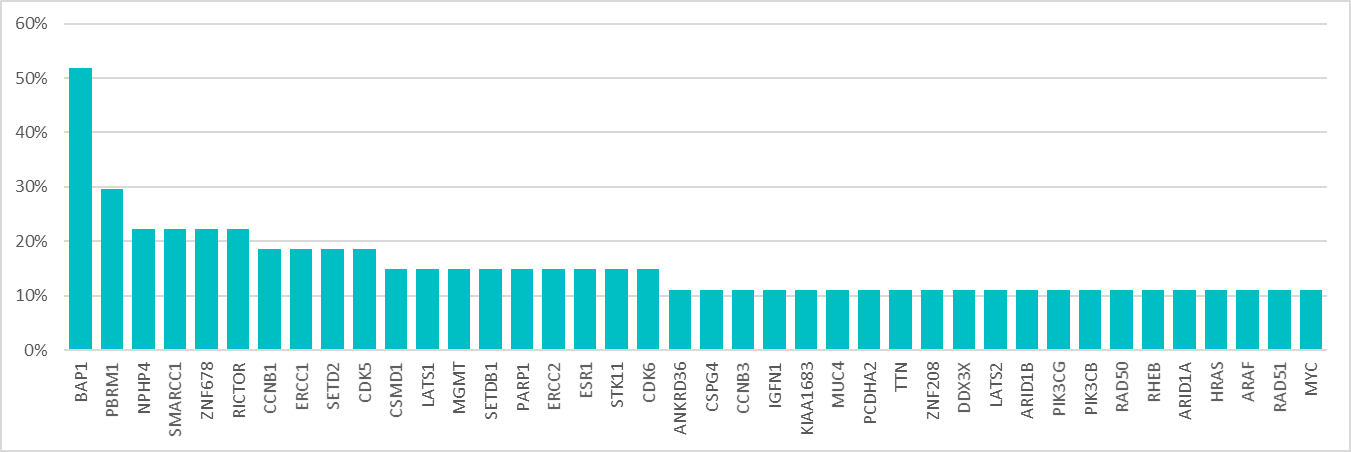


**Supplementary table 3.** Available targeted drugs approved by the EMA for other malignancies or available via the DRUP trial

| Targeted drug | Type of targeted therapy | Target gene | Approved indication by EMA | Indications DRUP study |
| --- | --- | --- | --- | --- |
| Abemaciclib | CDK4/6 inhibitors | CDK6 (amp)  CDK4 (amp)  CCND1 (amp) | HR-positive and HER2-negative advanced or metastatic breast cancer | CCND1 (amp): HNSCC, melanoma, NSCLSC, SCC bladder, ovarian, prostate, uracil, and urinary tract cancer  CDK4 (amp): glioblastoma, sarcoma, carcinoma of the small intestine, melanoma |
| Palbociclib | CDK4/6 inhibitors | CDK6 (amp)  CDK4 (amp)  CCND1 (amp)  *CDKN2A | HR-positive and HER2-negative advanced or metastatic breast cancer | CCND1 (amp): melanoma, NET, NSCLC, vulva carcinoma  CDK4 (amp): astrocytoma, esophageal cancer, PEComa, prostate cancer, sarcoma  CDK6 (amp): prostate cancer  CDKN2A (loss/mut): biliary tract, CRC, duodenal cancer, endometrial carcinoma, esophageal cancer, glioblastoma, HNSCC, melanoma, NET, NSCLC, (uvual) melanoma, ovarian cancer, pancreas cancer, primitive neuro-ectodermal tumor, RCC, vulva carcinoma |
| Ribociclib | CDK4/6 inhibitors | CDK4 (amp)  *CDKN2A | HR-positive and HER2-negative advanced or metastatic breast cancer | CDK4 (amp): melanoma  CDK6 (amp): MEC  CDKN2A (loss/mut): anaplastic meningioma, ACUP, ceruminous adenocarcinoma, ependymoma, gastric cancer, **mesothelioma**, prostate, salivary gland, sarcoma, thymus, urinary tract |
| Alpelisib | PI3K inhibitors | PIK3CA | HR-positive and HER2-negative advanced or metastatic breast cancer and a mutation in PIK3CA | PIK3CA (mut): gynecological (SCC/PCC), HNSCC, miscellaneous tumors, prostate cancer, upper-GI cancer. |
| Olaparib | PARP inhibitors | BRCA2  CHEK2  RAD50 | High-grade ovarian cancer, fallopian tubes or peritoneum and a mutation in BRCA1/2 or HRD positive; HER2-negative breast, or pancreatic, or prostate cancer, and a mutation in BRCA1/2; | BRCA1/2 (mut/loss): **tumor-agnostic**  HRR alterations: **all tumor types** |
| Talazoparib | PARP inhibitors | CHEK2 | HER2-negative breast cancer and a mutation in BRCA1/2 | HRD signature ^1^: **tumor-agnostic** |
| Rucaparib | PARP inhibitors | BRCA2 | High-grade cancer of ovary, fallopian tubes or peritoneum | N/A |
| Alectinib | ALK inhibitors | ALK (fus) | ALK-positive advanced NSCLC | ALK (fus): tumor-agnostic |
| Crizotinib | ALK inhibitors | ALK (fus)  MET | ALK- or ROS1-positive advanced NSCLC | MET (amp): CRC, esophageal cancer, gastric cancer, HCC, NSCLC, ovarian cancer  MET (mut): NSCLC, RCC  MET (fus): thyroid cancer  ALK (fus): sarcoma, ACUP, inflammatory myofibrolastic tumor |
| Lorlatinib | ALK inhibitors | ALK (fus) | ALK-positive advanced NSCLC | N/A |
| Trametinib | MEK inhibitors | GNAS (mut) | Advanced melanoma or NSCLC and a BRAF V600 mutation | GNAS (mut): **all tumor types** |
| Cabozantinib | Multi-targeted receptor tyrosine kinase inhibitors | MET (amp/fus) | Advanced RCC, HCC and differentiated thyroid carcinoma | MET (amp): teratoma, melanoma  MET (mut): NSCLC, esophageal cancer |
| Sotorasib | KRAS inhibitors | KRAS (G12C mut) | Advanced NSCLC and a KRAS G12C mutation | N/A |

ACUP= adenocarcinomas of unknown primary, amp= amplification, CRC= colorectal cancer, GIST= gastrointestinal stromal tumor, fus= fusion, HCC= hepatocellular carcinoma, HNSCC= head and neck squamous cell carcinoma, HR= hormone receptor, HRD= homologous recombination deficiency, HRR= homologous recombination repair, MEC= mucoepidermoid cancer, mut= mutation, NET= neuroendocrine tumor, NSCLC= non-small-cell lung cancer, PCC= primary peritoneal cancer, PEComa= perivascular epithelioid cell tumor, RCC= renal cell carcinoma, SCC= squamous cell carcinoma

^1^ Abbreviations in HRR genes like ARID1A, BAP1, BRCA2, BRIP1, CHEK2, RAD50, RAD51

**Supplementary table 4.** Trials investigating targeted therapies per identified gene (i.e. aberrations present in ≥10% of PeM patients)

| **Type(s) of targeted therapy** | **Drug(s)** | **Solid malignancy** | **Trial number** | **Phase** | **Status** | **Link** |
| --- | --- | --- | --- | --- | --- | --- |
| **Gene: BAP1** | | | | | | |
| EZH2 Inhibitor | Tazemetostat | Mesothelioma | NCT02860286 | 2 | Completed | https://clinicaltrials.gov/ct2/show/NCT02860286 |
| PARP inhibitor | Niraparib | Metastatic Breast Cancer | NCT04508803 | 2 | Not yet recruiting | <https://clinicaltrials.gov/show/NCT04508803> |
| PARP inhibitor | Niraparib | DDR Deficient Neoplasms | NCT03207347 | 2 | Active, not recruiting | https://clinicaltrials.gov/show/NCT03207347 |
| PARP inhibitor | Olaparib | Metastatic PDAC | NCT04666740 | 2 | Recruiting | https://clinicaltrials.gov/show/NCT04666740 |
| PARP inhibitor | Niraparib | Malignant Solid Tumor | NCT03209401 | 1 | Recruiting | https://clinicaltrials.gov/show/NCT03209401 |
| EZH2 Inhibitor | CPI-0209 | Advanced Tumors | NCT04104776 | 1/2 | Recruiting | https://clinicaltrials.gov/show/NCT04104776 |
| PARP inhibitor | Olaparib | Advanced Melanoma | NCT04633902 | 2 | Recruiting | <https://clinicaltrials.gov/show/NCT04633902> |
| PARP inhibitor | Olaparib | HRD Mesothelioma | NCT04515836 | 2 | Recruiting | https://clinicaltrials.gov/show/NCT04515836 |
| PARP inhibitor | Talazoparib | Melanoma | NCT04187833 | 2 | Recruiting | https://clinicaltrials.gov/show/NCT04187833 |
| PARP inhibitor | Niraparib | Melanoma | NCT03925350 | 2 | Recruiting | <https://clinicaltrials.gov/show/NCT03925350> |
| PARP inhibitor | Olaparib | Urothelial Cancer | NCT03375307 | 2 | Recruiting | https://clinicaltrials.gov/show/NCT03375307 |
| PARP inhibitor | Veliparib | Stage IV Solid Tumors | NCT03061188 | 1 | Active, not recruiting | https://clinicaltrials.gov/show/NCT03061188 |
| ATR inhibitor | M6620 | DDR Deficient Solid Tumors | NCT04266912 | 1/2 | Recruiting | https://clinicaltrials.gov/show/NCT04266912 |
| PARP inhibitor | Olaparib | Mesothelioma | NCT03531840 | 2 | Completed | https://clinicaltrials.gov/show/NCT03531840 |
| PARP inhibitor | Olaparib | Metastatic RCC | NCT03786796 | 2 | Recruiting | <https://clinicaltrials.gov/show/NCT03786796> |
| EZH2 Inhibitor | GSK2816126 | Various malignancies | NCT02082977 | 1 | Terminated | <https://clinicaltrials.gov/show/NCT02082977> |
| **Gene: NF2** | | | | | | |
| MEK inhibitor | Selumetinib | NF Type 2 Related Tumors | NCT03095248 | 2 | Recruiting | https://clinicaltrials.gov/show/NCT03095248 |
| VEGF inhibitor | Axitinib | NF Type 2 Related Tumors | NCT02129647 | 2 | Completed | <https://clinicaltrials.gov/show/NCT02129647> |
| EGFR/HER2 inhibitor | Lapatinib | VS | NCT00863122 | 1 | Completed | https://clinicaltrials.gov/show/NCT00863122 |
| mTOR inhibitor | Everolimus | NF2-related VS | NCT01345136 | 2 | Active, not recruiting | https://clinicaltrials.gov/show/NCT01345136 |
| Hh pathway inhibitor FAK Inhibitor | Vismodegib GSK2256098 | Progressive Meningiomas | NCT02523014 | 2 | Suspended | https://clinicaltrials.gov/show/NCT02523014 |
| TEAD inhibitor | VT3989 | Metastatic Solid Tumors | NCT04665206 | 1 | Recruiting | https://clinicaltrials.gov/show/NCT04665206 |
| HDAC Inhibitor | AR-42 | VS and Meningioma | NCT02282917 | 1 | active, not recruiting | <https://clinicaltrials.gov/show/NCT02282917> |
| NEDD8 inhibitor | Pevonedistat | Mesothelioma | NCT03319537 | 1/2 | Recruiting | https://clinicaltrials.gov/show/NCT03319537 |
| mTOR inhibitor | AZD2014 (Vistusertib) | Meningiomas | NCT02831257 | 2 | Completed | https://clinicaltrials.gov/show/NCT02831257 |
| MEK inhibitor | Mirdametinib | Low-Grade Glioma | NCT04923126 | 1/2 | Recruiting | <https://clinicaltrials.gov/show/NCT04923126> |
| mTOR inhibitor | Everolimus | Advanced Solid Tumors | NCT02352844 | 2 | Completed | <https://clinicaltrials.gov/show/NCT02352844> |
| mTOR inhibitor | Rapamycin | Advanced Cancer | NCT02646319 | 1 | Completed | https://clinicaltrials.gov/show/NCT02646319 |
| MEK inhibitor | Trametinib | Malignant Solid Tumor | NCT01827384 | 2 | Active, not recruiting | https://clinicaltrials.gov/show/NCT01827384 |
| Chk1 inhibitor | SRA737 | Advanced Cancer | NCT02797964 | 1/2 | Completed | https://clinicaltrials.gov/show/NCT02797964 |
| ATR inhibitor | M6620 | Advanced Solid Tumors | NCT02157792 | 1 | Completed | https://clinicaltrials.gov/show/NCT02157792 |
| **Gene: SETD2** | | | | | | |
| Wee1-kinase inhibitor | AZD1775 | Advanced Solid Tumors | NCT03284385 | 2 | Recruiting | https://clinicaltrials.gov/show/NCT03284385 |
| **Gene: TP53** | | | | | | |
| PARP inhibitor Wee1-kinase inhibitor | Olaparib  AZD1775 | Advanced Solid Tumors | NCT02576444 | 2 | Active, not recruiting | <https://clinicaltrials.gov/show/NCT02576444> |
| Angiogenesis inhibitor | Nintedanib | NSCLC | NCT02299141 | 2 | Active, not recruiting | <https://clinicaltrials.gov/show/NCT02299141> |
| VEGFR inhibitor | Pazopanib | Advanced NSCLC | NCT02193152 | 1 | Terminated | <https://clinicaltrials.gov/show/NCT02193152> |
| KIF18A inhibitor | AMG 650 | Advanced Solid Tumors | NCT04293094 | 1 | Recruiting | <https://clinicaltrials.gov/show/NCT04293094> |
| Wee1-kinase inhibitor | Adavosertib | Ovarian, Fallopian Tube, or Peritoneal Cancer | NCT02272790 | 2 | Active, not recruiting | <https://clinicaltrials.gov/show/NCT02272790> |
| Proteasome inhibitor HDAC inhibitor | MLN9708 Vorinostat | Advanced p53 Mutant Malignancies | NCT02042989 | 1 | Active, not recruiting | <https://clinicaltrials.gov/show/NCT02042989> |
| p53 activator | APR-246 | Recurrent HGSOC | NCT02098343 | 1/2 | Completed | <https://clinicaltrials.gov/show/NCT02098343> |
| **Gene: CDKN2A/B** | | | | | | |
| CDK4/6 inhibitor | Abemaciclib | [HNSCC](https://www.mycancergenome.org/content/disease/head-and-neck-squamous-cell-carcinoma/) | NCT03356223 | 2 | Recruiting | <https://clinicaltrials.gov/show/NCT03356223> |
| Aurora kinase inhibitor | Ilorasertib | Malignant Solid Tumor | NCT02478320 | 2 | Active, not recruiting | <https://clinicaltrials.gov/show/NCT02478320> |
| Aurora kinase inhibitor | Ilorasertib | Malignant Solid Tumor | NCT02540876 | 1 | Completed | <https://clinicaltrials.gov/show/NCT02540876> |
| CDK 4/6 inhibitor | Palbociclib Abemaciclib | Advanced Stage Cancer | NCT02693535 | 2 | Recruiting | <https://clinicaltrials.gov/show/NCT02693535> |
| CDK 4/6 inhibitor EGFR inhibitor | Palbociclib Cetuximab | [HNSCC](https://www.mycancergenome.org/content/disease/head-and-neck-squamous-cell-carcinoma/) | NCT04966481 | 3 | Not yet recruiting | <https://clinicaltrials.gov/show/NCT04966481> |
| CDK4/6 inhibitor | Abemaciclib | Glioblastoma | NCT04118036 | 2 | Suspended | https://clinicaltrials.gov/show/NCT04118036 |
| CDK4/6 inhibitor MDM-2 inhibitor | Ribociclib Siremadlin | Malignant Solid Tumor | NCT04116541 | 2 | Recruiting | <https://clinicaltrials.gov/show/NCT04116541> |
| MAT2A inhibitor | AG-270 | Advanced Solid Tumors or Lymphoma | NCT03435250 | 1 | Recruiting | <https://clinicaltrials.gov/show/NCT03435250> |
| CDK4/6 inhibitor | Abemaciclib | Glioblastoma | NCT04074785 | 1 | Recruiting | <https://clinicaltrials.gov/show/NCT04074785> |
| CDK 4/6 inhibitor | Palbociclib | Chordoma | NCT03110744 | 2 | Recruiting | <https://clinicaltrials.gov/show/NCT03110744> |
| CDK 4/6 inhibitor | Palbociclib | Melanoma | NCT03454919 | 2 | Unknown | <https://clinicaltrials.gov/show/NCT03454919> |
| CDK4/6 inhibitor | Ribociclib | CDK4/6 Pathway Activated Tumors | NCT02187783 | 2 | Completed | <https://clinicaltrials.gov/show/NCT02187783> |
| CDK4/6 inhibitor MEK inhibitor | Ribociclib Trametinib | Melanoma | NCT02645149 | 2 | Not yet recruiting | <https://clinicaltrials.gov/show/NCT02645149> |
| CDK4/6 inhibitor | Abemaciclib | Glioblastoma | NCT02981940 | 2 | Recruiting | <https://clinicaltrials.gov/show/NCT02981940> |
| PARP inhibitor ATR inhibitor | Olaparib  Ceralasertib | [Malignant Solid Tumor](https://www.mycancergenome.org/content/disease/malignant-solid-tumor/) | NCT02576444 | 2 | Active, not recruiting | <https://clinicaltrials.gov/show/NCT02576444> |
| CDK 4/6 inhibitor | Palbociclib | [Malignant Solid Tumor](https://www.mycancergenome.org/content/disease/malignant-solid-tumor/) | NCT03297606 | 2 | Recruiting | <https://clinicaltrials.gov/show/NCT03297606> |
| CDK 4/6 inhibitor | PD-0332991 | Melanoma | NCT02202200 | 1/2 | Unknown | <https://clinicaltrials.gov/show/NCT02202200> |
| CDK 4/6 inhibitor | Palbociclib | Malignant Solid Tumor | NCT02896335 | 2 | Recruiting | <https://clinicaltrials.gov/show/NCT02896335> |
| CDK4/6 inhibitor | Ribociclib | Malignant Glioma, Meningioma | NCT02933736 | 1 | Recruiting | <https://clinicaltrials.gov/show/NCT02933736> |
| CDK4/6 inhibitor mTOR inhibitor | Ribociclib Everolimus | Malignant Glioma | NCT03834740 | 1 | Recruiting | <https://clinicaltrials.gov/show/NCT03834740> |
| CDK4/6 inhibitor | Abemaciclib | Bone and Soft Tissue Sarcoma | NCT04040205 | 2 | Recruiting | <https://clinicaltrials.gov/show/NCT04040205> |
| ERK inhibitor CDK4/6 inhibitor | LY3214996 Abemaciclib | Malignant Glioma | NCT04391595 | 1 | Recruiting | <https://clinicaltrials.gov/show/NCT04391595> |
| CDK4/6 inhibitor | Abemaciclib | [HNSCC](https://www.mycancergenome.org/content/disease/head-and-neck-squamous-cell-carcinoma/) | NCT03356587 | 2 | Unknown | <https://clinicaltrials.gov/show/NCT03356587> |
| CDK4/6 inhibitor | Abemaciclib | [Malignant Solid Tumor](https://www.mycancergenome.org/content/disease/malignant-solid-tumor/) | NCT03994796 | 2 | Recruiting | <https://clinicaltrials.gov/show/NCT03994796> |
| CDK 4/6 inhibitor | Palbociclib | NSCLC | NCT02664935 | 2 | Recruiting | <https://clinicaltrials.gov/show/NCT02664935> |

DDR= DNA Damage Response, HGSOC=High Grade Serous Ovarian Cancer, HNSCC=Head and Neck Squamous Cell Carcinoma, HRD= Homologous Recombination Deficiency, NSCLC= Non-Small Cell Lung Carcinoma, PDAC= Pancreatic Ductal Adenocarcinoma, RCC= Renal Cell Carcinoma, VS=Vestibular Schwannoma.
